# The First Quack in America

**Published:** 1869-03

**Authors:** 


The First Quack in America.—Perhaps the first mention of a quack is found in the Massachusetts court records for 1631, which show that one Nicholas Knapp was sentenced to be fined and whipped "for taking upon him to cure the scurvy by a water of noe worth or value, which he sold att a very deare rate."

